# Cervical sagittal balance after consecutive three-level hybrid surgery versus anterior cervical discectomy and fusion: radiological results from a single-center experience

**DOI:** 10.1186/s13018-023-03819-0

**Published:** 2023-05-10

**Authors:** Shihao Chen, Yuxiao Deng, Hao Liu, Tingkui Wu, Kangkang Huang, Junbo He, Beiyu Wang

**Affiliations:** Department of Orthopedics, Orthopedic Research Institute, West China Hospital, Sichuan University, 37 Guoxue Lane, Chengdu, 610041 Sichuan China

**Keywords:** Cervical sagittal balance, Radiological outcomes, Hybrid surgery, Anterior cervical discectomy and fusion, Three-level surgery

## Abstract

**Introduction:**

According to the different numbers and relative locations of cervical disc replacement (CDR) and anterior cervical discectomy and fusion (ACDF), three-level hybrid surgery (HS) has many constructs. The purpose of this retrospective study was to compare the sagittal alignment parameters of HS and ACDF for cervical degenerative disc disease (CDDD) and the association of the respective parameters.

**Methods:**

This study involved patients with three-level CDDD who underwent ACDF or HS at our institution between June 2012 and August 2021. This follow-up included one-level CDR and two-level ACDF (type I group), two-level CDR and one-level ACDF (type II group) and three-level ACDF. Cervical sagittal alignment parameters included cervical lordosis (CL), segment alignment (SA), T1 slope (T1S), C2–C7 sagittal vertical axis (SVA), T1S-CL, C2 slope (C2S), occipital to C2 angle (O-C2A) and segment range of motion (ROM). Postoperative complications included adjacent segment degeneration, imbalance, prosthetic subsidence and heterotopic ossification.

**Results:**

The three groups with a total of 106 patients were better matched in terms of demographics. Patients who underwent HS had significantly higher CL than those who underwent ACDF at 1 week, 6 months, 12 months and the final follow-up after surgery, as well as significantly better SA at 12 months and the final follow-up. There was no significant difference in T1S, SVA, T1S-CL, C2S, O-C2A or segment ROM among the three groups after surgery. The T1S-CL was significantly associated with C2S in the type I and type II groups at the preoperative and final follow-up. There was no significant difference in postoperative complications among the three groups.

**Conclusions:**

Most improvements in cervical sagittal alignment (CL, SA, T1S, SVA, T1S-CL, C2S, O-C2A, and segmental ROM) were observed in all three groups postoperatively. HS was more advantageous than ACDF in the maintenance of postoperative CL and SA. Thus, three-level HS may be better for maintaining cervical curvature. The number of replacement segments differed in those who underwent HS but did not affect the correlation between T1S-CL and C2S, both of which are well balanced.

## Introduction

Anterior cervical discectomy fusion (ACDF) is generally accepted as the standard surgical treatment for cervical degenerative disc disease (CDDD) because of its excellent postoperative results. However, it may lead to kinematic and biomechanical changes in the adjacent segments, which may result in accelerated adjacent segment degeneration (ASD). Cervical disc replacement (CDR) has become increasingly prevalent in recent years as an alternative approach aimed primarily at preserving segmental range of motion (ROM) and reducing the risk of ASD. Recent years, hybrid surgery (HS) combining ACDF and CDR is increasingly being used for multilevel CDDD, which allows the optimal surgical approach to be tailored to each target segment based on the status of cervical disc degeneration. Although many studies have demonstrated that HS is a safe and effective surgical approach for the treatment of CDDD [1–4], few studies on multilevel surgeries of the cervical spine exist [5].

Cervical alignment plays an important role in compensating for spinal balance, transmitting axial loads, and maintaining mechanical function [6]. The study of cervical sagittal alignment began with normative data and expanded to include correlations with overall sagittal balance, prognosis in various conditions, surgical outcomes, and classification of cervical deformities, and prediction of ideal goals for cervical spine reconstruction [7]. Various imaging parameters have been proposed for the assessment of cervical spine alignment. Therefore, it is crucial to maintain cervical sagittal alignment after HS, whereas most studies have concentrated only on cervical lordosis (CL) [8, 9]. Other important cervical sagittal alignment parameters, including the C2–C7 sagittal vertical axis (SVA), T1 slope (T1S), T1 slope minus cervical lordosis (T1S-CL), C2 slope (C2S), and occipital to C2 angle (O-C2A), have been rarely studied [10]. At present, there is a lack of clinical evidence on whether they can be well maintained in multilevel HS.

The purpose of this study was to compare the sagittal alignment of consecutive patients undergoing three-level HS and ACDF and to investigate whether better sagittal parameters can be maintained after three-level HS surgery and the association of the respective parameters.

## Materials and methods

### Participants and procedure selection

A retrospective study was conducted involving patients with three-level CDDD who underwent ACDF or HS in our hospital between June 2012 and August 2021. The patients were divided into one-level CDR and two-level ACDF (type I group), two-level CDR and one-level ACDF (type II group) and three-level ACDF group [11]. The inclusion criteria consisted of (1) a diagnosis of cervical myelopathy and radiculopathy; (2) refractory to conservative treatments for at least 6 weeks; (3) lesion segment confirmed by clinical symptoms and imaging (computed tomography (CT), magnetic resonance imaging (MRI), and X-rays); and (4) surgery on three levels between C3 and C7 [11]. The exclusion criteria consisted of (1) previous surgery at the cervical spine or (2) the existence of cervical stenosis, osteoporosis, tumor, and infection. ACDF or CDR is selected according to the degree of degeneration in each segment. The indications of CDR at the lesion segment were according to previous studies, which were without instability (sagittal plane translation > 3 mm and sagittal plane angulation > 11°), without an absence of motion < 3°, without a disc height loss > 50%, and without facet joint degeneration [11]. If instability, bridging osteophytes, and facet degeneration were observed in the radiological images, ACDF was performed. Ethical approval was provided by the medical ethics committee of our hospital (No. 2019-567). All patients provided written informed consent.

### Surgical technique

One senior spine surgeon performed all HS or ACDF surgeries in this study. The surgery was performed as previously described [11]. A standard Smith-Robinson approach was used to reveal the surgical segment. The normal anatomy is carefully preserved and identified in preparation for the next step of fusion or replacement. After discectomy decompression of all target segments, an appropriately sized Prestige-LP disc and channel are inserted into the endplate. The appropriate size Zero-P implant system was subsequently inserted at the ACDF level and filled with β-tricalcium phosphate. C-arm fluoroscopy was performed to confirm the correct position of the implant. Finally, the incision was closed after insertion of the drainage tube.

### Data collection

The data were collected preoperatively and at 1 week, 3 months, 6 months, and 12 months postoperatively and at the final follow-up. Perioperative parameters, including operative time and blood loss, were collected.

### Radiological evaluation

**Fig. 1:**
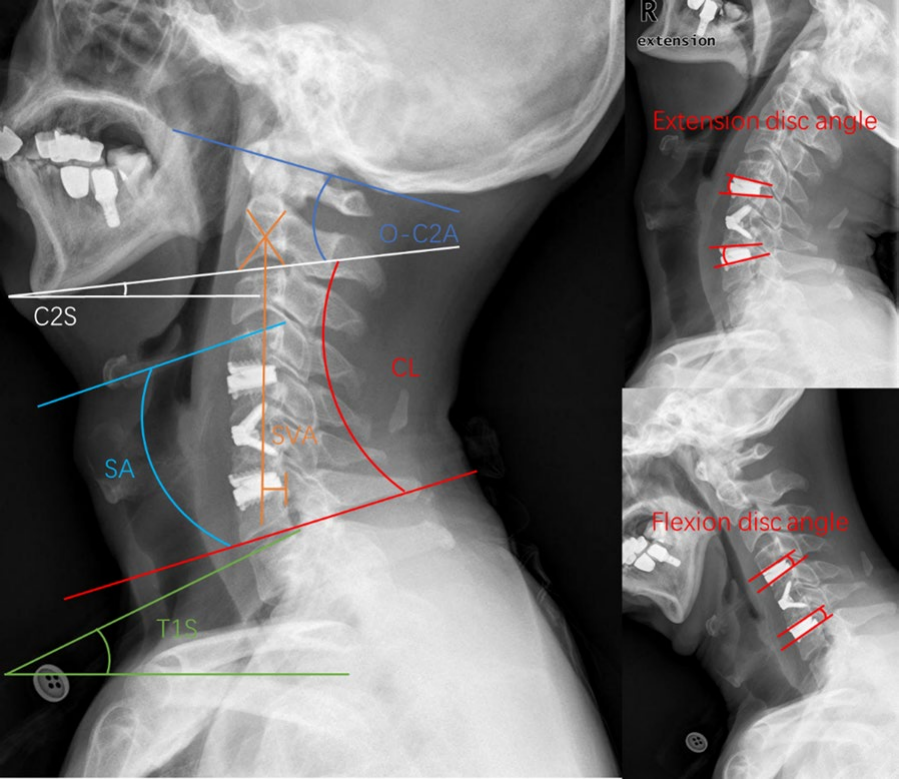
Schematic diagram of the parameters. X-rays show several cervical sagittal alignment parameters measured in this investigation. (1) CL; (2) SA; (3) T1S; (4) SVA; (5) T1S-CL; (6) C2S; (7) O-C2A; and (8) Segment ROM. *CL* cervical lordosis, *SA* segment alignment, *T1S* T1 slope, *SVA* sagittal vertical axis, *C2S* C2 slope, *O-C2A* occipital to C2 angle, *ROM* range of motion

Cervical sagittal alignment parameters were measured on the lateral radiographs. The following cervical sagittal alignment parameters were evaluated: (1) CL; (2) SA; (3) T1S; (4) SVA; (5) T1S-CL; (6) C2S; (7) O-C2A; and (8) Segment ROM. CL is the angle between the tangent line of the inferior endplate of the C2 and C7 vertebral bodies. SA is the angle between the tangent line of the superior and inferior endplates of the operative segment. T1S is the angle between the tangent line of the superior endplate of the T1 vertebral body and the horizontal line. C2-7 SVA is the horizontal distance between the plumb line of the geometric central C2 vertebral body and the posterior superior angle of the superior endplate of the C7 vertebral body [12]. T1S-CL is obtained by subtracting the previously determined C2–C7 lordosis angle from the T1 slope [13]. C2S is the angle between the tangent line of the inferior endplate of the C2 vertebral body and the horizontal line [14]. O-C2A is the angle between the McGregor line and the line connecting the inferior endplates of the C2 vertebral body. Segment ROM is recognized as the extension-flexion segment angle (Fig. 1).

ASD was defined based on the height of an adjacent level disc and anterior osteophyte formation on X-rays according to the classification reported by Goffin et al. [15]. T1S-CL was used to evaluate the cervical sagittal balance (T1S-CL < 15°, balance; T1S-CL ≥ 15°, imbalance) [12, 16]. Prosthetic subsidence was considered to be a change of > 5° in the tangential angle along the lower edge of the prosthesis to the posterior edge of the vertebral body between the final postoperative follow-up and 1 week postoperatively [17]. According to the McAfee classification criteria, heterotopic ossification (HO) was defined as the exposed bony end plates of the vertebral bodies at the surgical-level growth toward the artificial [18].

### Statistical analysis

All statistical analyses were performed using SPSS (version 24.0, SPSS, Chicago, IL, USA). Continuous variables are presented as the mean ± standard deviation (SD), and categorical variables are presented as the number of cases. ANOVA and Tukey tests were applied to compare the clinical and radiographic effects as qualitative data among the three groups. A paired t test was used to compare the clinical outcomes and sagittal alignment parameters pre- and postoperation. Student’s t test or the Mann‒Whitney *U* test was used to compare continuous variables depending on the normality of the data. A chi-square test or Fisher’s exact test was used to analyze categorical data. The correlations between sagittal alignment parameters were analyzed using the Pearson correlation coefficient. Statistical significance was defined as *p* < 0.05.

## Results

### Demographic and surgical data

A total of 106 patients were included in the analysis according to the inclusion and exclusion criteria, including 47 patients in the type I group, 26 patients in the type II group, and 33 patients in the ACDF group. There were no significant differences between the three groups in terms of sex ratio, body mass index (BMI), surgical level distribution, mean blood loss, or mean follow-up time. The mean age of patients in the ACDF group was significantly older than that in the type II group (*p* < 0.05). The operative time in the type II group was 174.39 min ± 23.72 min, which was significantly longer than that in the ACDF group (*p* < 0.05). However, there was no significant difference between the ACDF group and the type I group or the type I group and the type II group. Detailed information is shown in Table 1.

**Table 1:** Summary of the patient demographic data

	Type I	Type II	ACDF	*p* value
N	47	26	33	
Gender, n				
Male	20	13	17	0.692^a^
Female	27	13	16	
Age, year	50.87 ± 8.20	47.12 ± 7.39	56.70 ± 12.59	**0.002**^**b**^
BMI	24.33 ± 3.47	24.80 ± 3.87	24.48 ± 3.28	0.862^b^
Levels, n				
C3–6	14	10	10	0.724^a^
C4–7	33	16	23	
Operation time, min	164.83 ± 20.78	174.39 ± 23.72	156.24 ± 28.38	**0.035**^**b**^
Blood loss, ml	70.64 ± 21.00	70.77 ± 18.96	67.27 ± 17.19	0.704^b^
FU, mouths	22.15 ± 13.61	21.50 ± 12.44	29.13 ± 9.3	0.378^b^

Bold values indicate statistically different

*ACDF* anterior cervical discectomy and fusion, *BMI* body mass index, *FU* follow-up

^a^Chi-square test for the three groups

^b^ANOVA test for the three groups

### Radiological outcomes

**Fig. 2:**
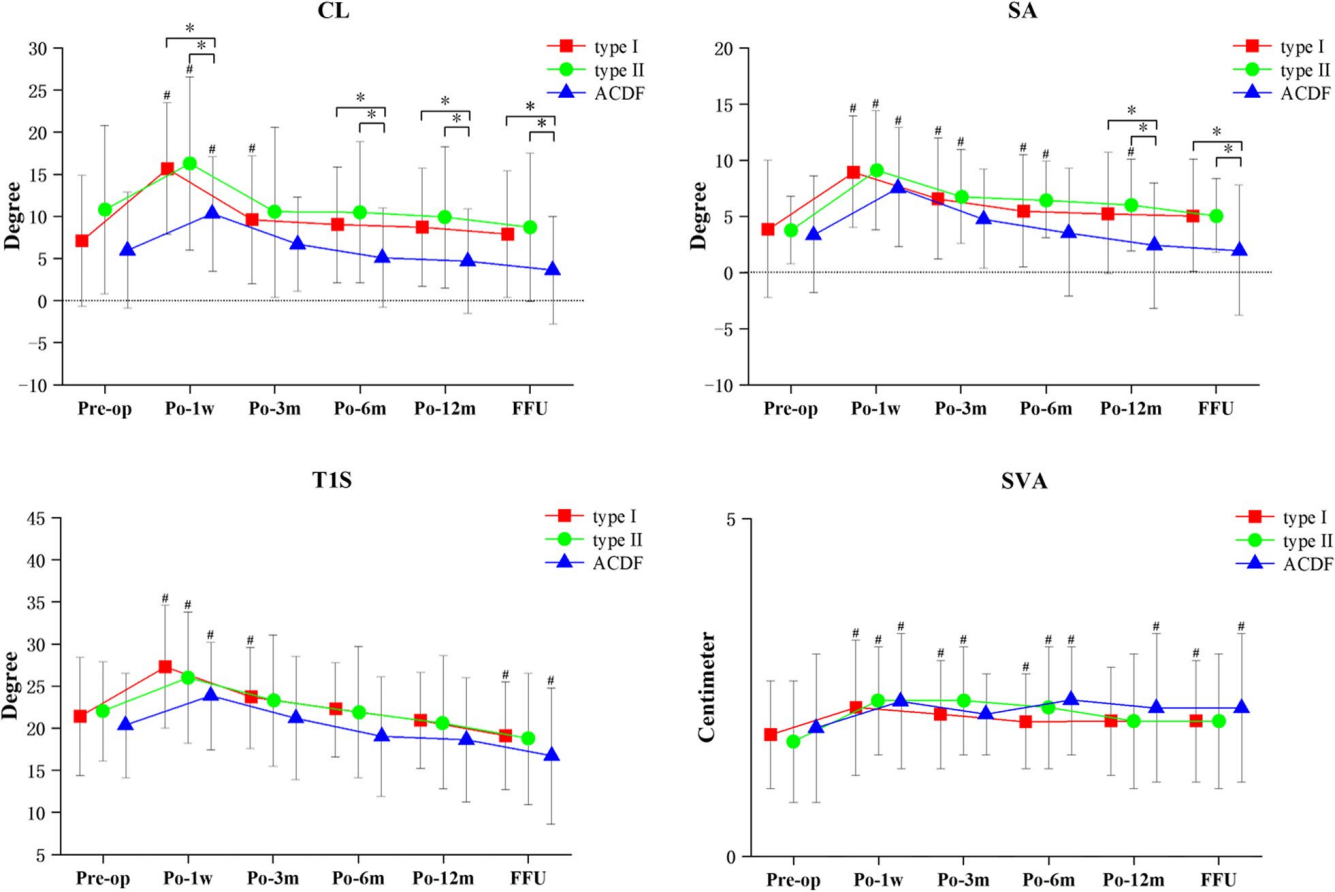
CL, SA, T1S and SVA. (#*p* < 0.05 significance on parameters between pre-op, *p < 0.05 between two groups). *Pre* preoperative, *Po* postoperative, *FFU* final follow-up, *CL* cervical lordosis, *SA* segment alignment, *T1S* T1 slope, *SVA* sagittal vertical axis

At 12 months postoperatively and at the final follow-up, CL and SA were significantly lesser in the ACDF group than in the type I and type II groups (*p* < 0.05). Furthermore, CL in the ACDF group was also significantly lesser than that in the type I and II groups at 1 week and 6 months postoperatively (*p* < 0.05). A week after surgery, CL, SA, T1S and SVA were significantly more pronounced in all three groups than in the preoperative period (*p* < 0.05). Furthermore, SA and SVA were significantly higher in both the type I and type II groups at 3 and 6 months postoperatively (*p* < 0.05). For the final follow-up, there was no statistically significant difference in CL and SA of the three groups when compared to the preoperative period. However, both the type I and ACDF groups showed a significant decrease at the final follow-up when compared to the preoperative values (*p* < 0.05) (Table 2) (Fig. 2).

**Table 2:** Summary of the patient radiological analysis

	Type I	Type II	ACDF	*p* value
*CL*				
Pre	7.1 ± 7.8	10.8 ± 10.0	6.0 ± 6.9	0.068^a^
Po-1w	15.7 ± 7.8#	16.3 ± 10.3#	10.3 ± 6.8#	**0.003**^**a**^
Po-3 m	9.6 ± 7.6#	10.5 ± 10.1	6.7 ± 5.6	0.087^a^
Po-6 m	9.0 ± 6.9	10.5 ± 8.4	5.1 ± 5.9	**0.009**^**a**^
Po-12 m	8.7 ± 7.0	9.9 ± 8.4	4.7 ± 6.2	**0.012**^**a**^
FFU	7.9 ± 7.5	8.7 ± 8.8	3.6 ± 6.4	**0.016**^**a**^
*SA*				
Pre	3.9 ± 6.1	3.8 ± 3.0	3.4 ± 5.2	0.899^a^
Po-1w	9.0 ± 5.0#	9.1 ± 5.3#	7.6 ± 5.3#	0.405^a^
Po-3 m	6.6 ± 5.4#	6.8 ± 4.2#	4.8 ± 4.4	0.179^a^
Po-6 m	5.5 ± 5.0#	6.5 ± 3.4#	3.6 ± 5.7	0.069^a^
Po-12 m	5.3 ± 5.4	6.0 ± 4.1#	2.4 ± 5.6	**0.013**^**a**^
FFU	5.1 ± 5.0	5.1 ± 3.3	2.0 ± 5.8	**0.015**^**a**^
*T1S*				
Pre	21.4 ± 7.0	22.0 ± 5.9	20.3 ± 6.2	0.583^a^
Po-1w	27.3 ± 7.3#	26.0 ± 7.8#	23.8 ± 6.4#	0.103^a^
Po-3 m	23.6 ± 6.0#	23.3 ± 7.8	21.2 ± 7.3	0.284^a^
Po-6 m	22.2 ± 5.6	21.9 ± 7.8	19.0 ± 7.1	0.091^a^
Po-12 m	20.9 ± 5.7	20.7 ± 7.9	18.6 ± 7.4	0.306^a^
FFU	19.1 ± 6.4a	18.7 ± 7.8	16.7 ± 8.1aa	0.334^a^
*SVA*				
Pre	1.8 ± 0.8	1.7 ± 0.9	1.9 ± 1.1	0.693^a^
Po-1w	2.2 ± 1.0#	2.3 ± 0.8#	2.3 ± 1.0#	0.915^a^
Po-3 m	2.1 ± 0.8#	2.3 ± 0.8#	2.1 ± 0.6	0.467^a^
Po-6 m	2.0 ± 0.7#	2.2 ± 0.9#	2.3 ± 0.8#	0.221^a^
Po-12 m	2.0 ± 0.8	2.0 ± 1.0	2.2 ± 1.1#	0.674^a^
FFU	2.0 ± 0.9#	2.0 ± 1.0	2.2 ± 1.1#	0.810^a^
*T1S-CL*				
Pre	14.3 ± 9.3	11.1 ± 10.7	14.3 ± 8.6	0.339^a^
Po-1w	11.6 ± 8.9	9.8 ± 10.9	12.7 ± 9.0	0.499^a^
Po-3 m	14.0 ± 7.7	12.8 ± 9.8	14.5 ± 8.6	0.741^a^
Po-6 m	13.1 ± 7.2	11.4 ± 8.1	13.9 ± 8.0	0.477^a^
Po-12 m	12.2 ± 7.7	10.8 ± 9.9	14.0 ± 8.7	0.369^a^
FFU	11.3 ± 8.9#	10.0 ± 9.5	13.1 ± 10.0	0.435^a^
*C2S*				
Pre	12.4 ± 7.2	12.0 ± 7.3	13.4 ± 6.7	0.726^a^
Po-1w	10.4 ± 7.3	9.3 ± 6.4	12.9 ± 6.3	0.109^a^
Po-3 m	12.2 ± 6.8	11.8 ± 7.0	12.7 ± 8.6	0.889^a^
Po-6 m	11.5 ± 6.2	11.0 ± 6.9	11.9 ± 7.6	0.885^a^
Po-12 m	9.5 ± 5.8#	9.5 ± 5.8	10.9 ± 6.8#	0.585^a^
FFU	8.3 ± 5.7#	8.4 ± 5.9#	10.5 ± 7.6#	0.258^a^
*O-C2A*				
Pre	20.9 ± 7.5	18.7 ± 7.0	20.5 ± 7.3	0.433^a^
Po-1w	16.6 ± 6.4#	16.7 ± 8.0	18.8 ± 5.6	0.272^a^
Po-3 m	20.3 ± 6.9	20.5 ± 9.3	21.9 ± 7.1	0.622^a^
Po-6 m	20.5 ± 6.8	19.6 ± 8.8	20.3 ± 6.7	0.871^a^
Po-12 m	19.6 ± 6.2	18.2 ± 8.4	19.6 ± 6.8	0.690^a^
FFU	18.2 ± 6.9#	17.2 ± 8.5	15.7 ± 5.1#	0.198^a^
*Segment ROM*				
Pre	12.2 ± 4.5	11.0 ± 4.9		0.205^b^
Po-1w	5.5 ± 3.9#	7.4 ± 5.1#		**0.036**^**b**^
Po-3 m	6.9 ± 3.9#	7.7 ± 4.4#		0.327^b^
Po-6 m	7.4 ± 4.3#	10.6 ± 4.5		**0.000**^**b**^
Po-12 m	8.8 ± 4.6#	10.9 ± 3.5		**0.014**^**b**^
FFU	9.2 ± 4.7#	12.6 ± 4.1		**0.000**^**b**^

Bold values indicate statistically different

*ACDF* anterior cervical discectomy and fusion, *Pre* preoperative, *Po* postoperative, *FFU* final follow-up, *CL* cervical lordosis, *SA* segment alignment, *T1S* T1 slope, *SVA* sagittal vertical axis, *C2S* C2 slope, *O-C2A* occipital to C2 angle, *ROM* range of motion

^#^Significance on parameters between pre-op (*p* < 0.05)

^a^ANOVA test for the three groups

^b^Independent-Samples T Test

**Fig. 3:**
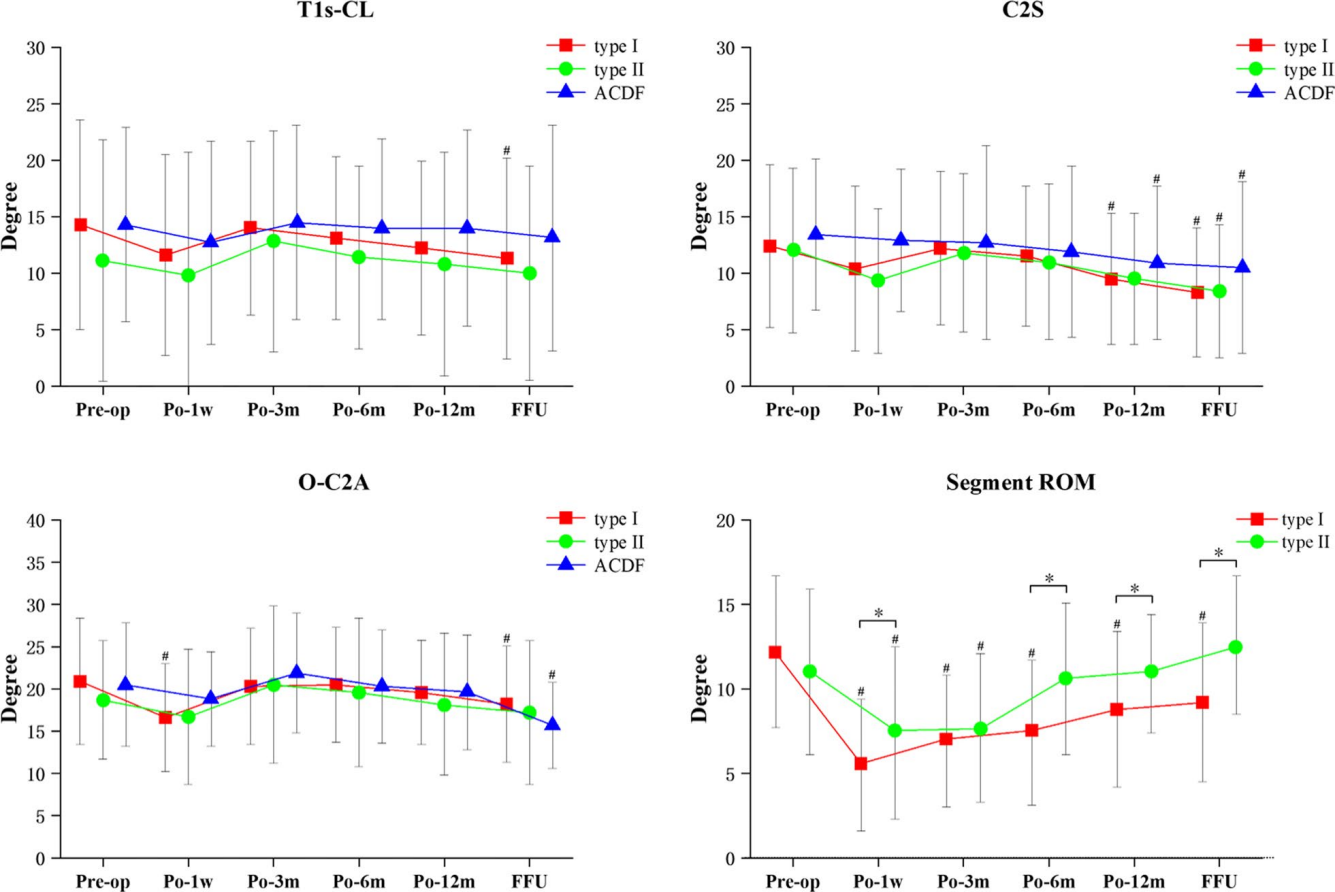
T1S-CL, C2S, O-C2A and Segment ROM. (#*p* < 0.05 significance on parameters between pre-op, *p < 0.05 between two groups). *Pre* preoperative, *Po* postoperative, *FFU* final follow-up, *T1S* T1 slope, *CL* cervical lordosis, *C2S* C2 slope, *O-C2A* occipital to C2 angle, *ROM* range of motion

Segment ROM was better at 1 week, 6 months, 12 months and the final follow-up after type II surgery than after type I surgery (*p* < 0.05). The T1S-CL, C2S, O-C2A and segment ROM for the patients in the type I group were all significantly lesser at the final follow-up when compared to the preoperative values (*p* < 0.05). Compared to the preoperative period, both type I of O-C2A and type I and type II of segment ROM were significantly decreased at 1 week postoperatively (*p* < 0.05). However, the other parameters decreased but were not significantly different. The final postoperative follow-up of C2S was significantly lesser in all three groups than that of the preoperative period (*p* < 0.05). At all postoperative follow-ups, the ROM of type I segments was significantly lesser than that preoperatively, whereas type II segments decreased significantly only at 1 week and 3 months postoperatively (Table 2) (Fig. 3).

#### Correlation between sagittal alignment parameters of type I

CL was significantly correlated with O-C2A (r_Pre_ = − 0.563; r_FFU_ = − 0.290, *p* < 0.05) and preoperative SVA (r_Pre_ = − 0.341, *p* < 0.05). T1S-CL was significantly correlated with CL (r_Pre_ = − 0.675; r_FFU_ = − 0.703, *p* < 0.05), T1S (r_Pre_ = 0.570; r_FFU_ = 0.566, *p* < 0.05), SVA (r_Pre_ = 0.532; r_FFU_ = 0.425, *p* < 0.05), C2S (r_Pre_ = 0.769; r_FFU_ = 0.471, *p* < 0.05) and O-C2A (r_Pre_ = 0.446; r_FFU_ = 0.324, *p* < 0.05). C2S was significantly correlated with CL (r_Pre_ = − 0.674; r_FFU_ = − 0.321, *p* < 0.05), SVA (r_Pre_ = 0.533; r_FFU_ = 0.338, *p* < 0.05) and O-C2A (r_Pre_ = 0.559; r_FFU_ = 0.393, *p* < 0.05). T1S was significantly correlated with SVA (r_Pre_ = 0.323; r_FFU_ = 0.290, *p* < 0.05) (Table 3).

**Table 3:** Correlation between sagittal alignment parameters of type I

	CL	SA	T1S	SVA	T1S-CL	C2S	O-C2A
*Pre*							
CL	1	**0.617#**	0.222	**− 0.341#**	**− 0.675#**	**− 0.674#**	**− 0.563#**
SA		1	**0.338#**	− 0.147	− 0.264	**− 0.426#**	**− 0.554#**
T1S			1	**0.323#**	**0.570#**	0.266	− 0.038
SVA				1	**0.532#**	**0.533#**	0.219
T1S-CL					1	**0.769#**	**0.446#**
C2S						1	**0.559#**
O-C2A							1
*FFU*							
CL	1	**0.421#**	0.188	− 0.256	**− 0.703#**	**− 0.321#**	**− 0.290#**
SA		1	0.189	0.066	− 0.216	0.034	− 0.070
T1S			1	**0.290#**	**0.566#**	0.279	0.111
SVA				1	**0.425#**	**0.338#**	0.187
T1S-CL					1	**0.471#**	**0.324#**
C2S						1	**0.393#**
O-C2A							1

Bold values indicate statistically different

*Pre* preoperative, *FFU* final follow-up, *CL* cervical lordosis, *SA* segment alignment, *T1S* T1 slope, *SVA* sagittal vertical axis, *C2S* C2 slope, *O-C2A* occipital to C2 angle

^#^Significant correlation between parameters (*p* < 0.05)

#### Correlation between sagittal alignment parameters of type II

CL was significantly correlated with T1S-CL (r_Pre_ = − 0.840; r_FFU_ = − 0.635, *p* < 0.05), C2S (r_Pre_ = − 0.463; r_FFU_ = − 0.626, *p* < 0.05) and O-C2A (r_Pre_ = − 0.536; r_FFU_ = − 0.450, *p* < 0.05). T1S-CL was significantly correlated with T1S (r_Pre_ = 0.395; r_FFU_ = 0.495, *p* < 0.05), T1S-CL (r_Pre_ = 0.515; r_FFU_ = 0.531, *p* < 0.05)**,** final follow-up SVA (r_FFU_ = 0.462, *p* < 0.05) and final follow-up O-C2A (r_FFU_ = 0.495, *p* < 0.05). C2S was significantly correlated with final follow-up O-C2A (r_FFU_ = 0.622, *p* < 0.05) (Table 4).

**Table 4:** Correlation between sagittal alignment parameters of type II

	CL	SA	T1S	SVA	T1S-CL	C2S	O-C2A
*Pre*							
CL	1	**0.394#**	0.166	− 0.166	**− 0.840#**	**− 0.463#**	**− 0.536#**
SA		1	0.042	− 0.144	− 0.344	− 0.091	− 0.192
T1S			1	− 0.027	**0.395#**	0.153	− 0.382
SVA				1	0.140	0.373	0.010
T1S-CL					1	**0.515#**	0.289
C2S						1	0.376
O-C2A							1
*FFU*							
CL	1	0.182	0.357	− 0.243	**− 0.635#**	**− 0.626#**	**− 0.450#**
SA		1	0.115	− 0.238	− 0.074	− 0.025	− 0.064
T1S			1	0.285	**0.495#**	− 0.063	0.092
SVA				1	**0.462#**	0.343	0.173
T1S-CL					1	**0.531#**	**0.495#**
C2S						1	**0.622#**
O-C2A							1

Bold values indicate statistically different

*Pre* preoperative, *FFU* final follow-up, *CL* cervical lordosis, *SA* segment alignment, *T1S* T1 slope, *SVA* sagittal vertical axis, *C2S* C2 slope, *O-C2A* occipital to C2 angle

^#^Significant correlation between parameters (*p* < 0.05)

#### Correlation between sagittal alignment parameters of type I and type II

CL was significantly correlated with SVA (r_Pre_ = − 0.265; r_FFU_ = − 0.250, *p* < 0.05), T1S-CL (r_Pre_ = − 0.754; r_FFU_ = − 0.675, *p* < 0.05), C2S (r_Pre_ = − 0.575; r_FFU_ = − 0.440, *p* < 0.05) and O-C2A (r_Pre_ = − 0.560; r_FFU_ = − 0.363, *p* < 0.05). T1S-CL was significantly correlated with T1S (r_Pre_ = 0.491; r_FFU_ = 0.536, *p* < 0.05), SVA (r_Pre_ = 0.365; r_FFU_ = 0.439, *p* < 0.05), C2S (r_Pre_ = 0.664; r_FFU_ = 0.491, *p* < 0.05) and O-C2A (r_Pre_ = 0.400; r_FFU_ = 0.397, *p* < 0.05). C2S was significantly correlated with SVA (r_Pre_ = 0.467; r_FFU_ = 0.340, *p* < 0.05) and O-C2A (r_Pre_ = 0.493; r_FFU_ = 0.483, *p* < 0.05) (Table 5).

**Table 5:** Correlation between sagittal alignment parameters of type I and type II

	CL	SA	T1S	SVA	T1S-CL	C2S	O-C2A
*Pre*							
CL	1	**0.503#**	0.201	**− 0.265#**	**− 0.754#**	**− 0.575#**	**− 0.560#**
SA		1	**0.277#**	− 0.135	**− 0.262#**	**− 0.339#**	**− 0.459#**
T1S			1	0.193	**0.491#**	0.228	− 0.146
SVA				1	**0.365#**	**0.467#**	0.144
T1S-CL					1	**0.664#**	**0.400#**
C2S						1	**0.493#**
O-C2A							1
*FFU*							
CL	1	**0.337#**	**0.260#**	**− 0.250#**	**− 0.675#**	**− 0.440#**	**− 0.363#**
SA		1	0.160	− 0.022	− 0.173	0.018	− 0.066
T1S			1	**0.288#**	**0.536#**	0.139	0.104
SVA				1	**0.439#**	**0.340#**	0.181
T1S-CL					1	**0.491#**	**0.397#**
C2S						1	**0.483#**
O-C2A							1

Bold values indicate statistically different

*Pre* preoperative, *FFU* final follow-up, *CL* cervical lordosis, *SA* segment alignment, *T1S* T1 slope, *SVA* sagittal vertical axis, *C2S* C2 slope, *O-C2A* occipital to C2 angle

^#^Significant correlation between parameters (*p* < 0.05)

## Summary of the patient complications

There was no significant difference in the incidence of postoperative ASD (incidence: 31.9%, 26.9%, 42.4%, respectively, *p* = 0.424) or imbalance (incidence: 29.8%, 23.1%, 36.4%, respectively, *p* = 0.542) between the patients included in the type I, type II or ACDF group. There was no significant difference the incidence of postoperative prosthetic subsidence (incidence: 17.0% and 23.1%, respectively, *p* = 0.750) or HO (incidence: 40.4% and 57.7%, respectively, p = 0.157) between the patients included in either the type I or type II group (Table 6).

**Table 6:** Summary of the patient complications

	Type I	Type II	ACDF	*p* value
N	47	26	33	
ASD	15	7	14	0.424^a^
Imbalance	14	6	12	0.542^a^
Prosthesis subsidence	8	6		0.750^b^
HO	19	15		0.157^b^

*ACDF* anterior cervical discectomy and fusion, *ASD* adjacent segment degeneration, *HO* heterotopic ossification

^a^Chi-square test for the three groups

^b^Chi-square test for the two groups

Of the 34 patients undergoing HS (46.6%) with HO observed at the final follow-up, 26.5% (n = 9), 29.4% (n = 10), 32.4% (n = 11) and 11.8% (n = 4) were McAfee grades I, II, III, and IV, respectively. Patients with and without HO showed equivalent changes in CL (8.7° vs. 7.7°; *p* = 0.599), SA (5.0° vs. 5.2°; p = 0.900), T1S (18.3° vs. 19.6°; *p* = 0.447), SVA (2.1 mm vs. 2.0 mm; *p* = 0.551), T1S-CL (9.6° vs. 11.8°; *p* = 0.297), C2S (8.3° vs. 8.3°; *p* = 0.974), O-C2A (17.1° vs. 18.4°; p = 0.457), segment ROM (10.3° vs. 11.6°; *p* = 0.158).

## Discussion

CDDD is a chronic, acquired deterioration of the cervical spine that can cause neck pain, radiculopathy, and/or myelopathy [19]. HS can be tailored to rebuild cervical stability at the target level, depending on the degree of degeneration of the different segments. Theoretically, an adequate range of motion is achieved at the level of joint replacement, and fixation is achieved at the level of joint fusion [20]. Cervical sagittal balance is associated with the development of cervical spine-related disorders and a decrease in health-related quality of life [21–23]. Therefore, it has become increasingly important to assess and correct cervical sagittal alignment during surgical treatment. In this study, we showed that multilevel HS surgery was superior to ACDF in maintaining CL and SA, while there was no difference between them in other parameters. This result may suggest that three-level hybrid surgery may be superior to ACDF in cervical sagittal alignment, and it may delay the development of ASD in the long term.

Cervical sagittal alignment has been a heavily debated and controversial issue. Xu et al. [24] compared three-level HS (Prodisc-C and MC+) and ACDF through more than 5 years of follow-up. Although most patients achieved cervical balance with HS and ACDF, no difference was found between the two surgery methods. In our previous research, we more accurately classified three-segment HS (Prestige-LP and Zero-P) as type I versus type II, and showed that type II (two-level CDR and one-level ACDF) was superior in terms of cervical lordosis and ROM [11]. The different prostheses and cages used may be the main factor in this difference. In the current study, compared to ACDF, multilevel HS also showed superior in maintaining cervical curvature, while similar cervical sagittal balance can be obtained in all other aspects [25, 26].

Multiple disorders of the cervical spine can lead to an imbalance in the sagittal alignment of the cervical spine [27, 28], and the linkage and interplay between cervical sagittal alignment parameters was demonstrated in this study. In both ACDF and HS, reconstruction of the CL is performed, making both cervical lordosis and T1S more pronounced. Both of these affect SVA simultaneously, with a greater CL causing a posterior shift of the head's center of gravity resulting in a lower SVA and a greater T1S causing an anterior shift of the head and cervical center of gravity resulting in a higher SVA. This is a compensatory mechanism to maintain horizontal gaze in response to changes in the overall sagittal alignment [29]. By showing that SVA is elevated after surgery in this retrospective study, the effect of T1S on SVA will be greater than the effect of CL on SVA, and the patient's head and cervical spine are anteriorly displaced after surgery.

C2S is a recently considered single, simplified measure of cervical deformity, similar to the T1S-CL measure. Shen et al. [30] showed that a greater C2S was associated with the presence of preoperative adjacent segmental pathology. C2S can adequately describe cervical deformity because of the association between O-C2A and CL, and both are closely related [31]. Similarly, a similar trend as well as a significant correlation between C2S and T1S-CL was observed in both type I and type II groups in this study. The results suggest that the different number of replacement segments in HS does not affect the correlation between T1S-CL and C2S, and both are well-balanced. Furthermore, this study also indicates that CL is negatively correlated with T1S-CL, C2S and O-C2A, while T1S is positively correlated with T1S-CL.

Due to the presence of both CDA and ACDF surgical segments in HS, the associated complications exhibit two distinct characteristics. The current study provides a summary of the typical complications experienced by patients. However, no statistically significant differences were observed, likely due to limitations in sample size. Several studies have failed to demonstrate a significant impact of HO on patient outcomes, and furthermore, HO did not influence postoperative cervical alignment [32, 33]. Hence, the clinical and radiographic implications of HO remain elusive. Although high HO rates do not significantly alter cervical sagittal parameters in the short term, they may pose a concern for disrupting cervical alignment in the long term, as suggested by the findings of this study. Therefore, it may be necessary to conduct large-scale studies with longer follow-up periods in order to gain a better understanding of how the loss of segmental mobility caused by HO affects cervical spine alignment.

This study has several limitations. Firstly, as a retrospective, single-center study, there may have been differences in surgery indications between groups and nonuniform baselines which could lead to potential biases. In addition, the mean age of the patients in each group varied, which may have biased the results. Second, the sample size was relatively small, especially for patients in the type II group undergoing HS, and the follow-up period was relatively short. Third, only the Zero-P and Prestige-LP systems were included in the study. In the future, prospective, multicenter, large-scale studies with different prostheses should be conducted to confirm these results. Fourth, the focus of this study was the radiological results of HS; therefore, its clinical outcomes were not considered.

## Conclusions

Most improvements in cervical sagittal alignment (CL, SA, T1S, SVA, T1S-CL, C2S, O-C2A, and segmental ROM) were observed in all three groups postoperatively. HS was more advantageous than ACDF in the maintenance of postoperative CL and SA. Thus, three-level HS may be better for maintaining cervical curvature. The number of replacement segments differed in those who underwent HS but did not affect the correlation between T1S-CL and C2S, both of which are well balanced.

## Data Availability

Datasets are available from the corresponding author on a reasonable request.

## Abbreviations

CDR: Cervical disc replacement
ACDF: Anterior cervical discectomy and fusion
HS: Hybrid surgery
CDDD: Cervical degenerative disc disease
BMI: Body mass index
CL: Cervical lordosis
SA: Segment alignment
T1S: T1 slope
SVA: Sagittal vertical axis
C2: C2 slope
O-C2A: Occipital to C2 angle
ROM: Segment range of motion
ASD: Adjacent segment degeneration
HO: Heterotopic ossification
TIS-CL: T1 slope minus cervical lordosis
